# The Mcm2-7 Replicative Helicase: A Promising Chemotherapeutic Target

**DOI:** 10.1155/2014/549719

**Published:** 2014-08-28

**Authors:** Nicholas E. Simon, Anthony Schwacha

**Affiliations:** Department of Biological Sciences, University of Pittsburgh, Pittsburgh, PA 15260, USA

## Abstract

Numerous eukaryotic replication factors have served as chemotherapeutic targets. One replication factor that has largely escaped drug development is the Mcm2-7 replicative helicase. This heterohexameric complex forms the licensing system that assembles the replication machinery at origins during initiation, as well as the catalytic core of the CMG (Cdc45-Mcm2-7-GINS) helicase that unwinds DNA during elongation. Emerging evidence suggests that Mcm2-7 is also part of the replication checkpoint, a quality control system that monitors and responds to DNA damage. As the only replication factor required for both licensing and DNA unwinding, Mcm2-7 is a major cellular regulatory target with likely cancer relevance. Mutations in at least one of the six *MCM *genes are particularly prevalent in squamous cell carcinomas of the lung, head and neck, and prostrate, and *MCM* mutations have been shown to cause cancer in mouse models. Moreover various cellular regulatory proteins, including the Rb tumor suppressor family members, bind Mcm2-7 and inhibit its activity. As a preliminary step toward drug development, several small molecule inhibitors that target Mcm2-7 have been recently discovered. Both its structural complexity and essential role at the interface between DNA replication and its regulation make Mcm2-7 a potential chemotherapeutic target.

## 1. Introduction

Misregulated DNA replication is a basic prerequisite for uncontrolled cellular proliferation, and the clinical targeting of eukaryotic replication factors has seen widespread use in cancer treatment. Small molecule inhibitors that predominantly target leading or lagging strand synthesis, such as topoisomerases [1], DNA polymerases [2], DNA ligase [3], proliferating cell nuclear antigen (PCNA) [4], ribonucleotide reductase [5], and telomerase [6], have been developed to clinically block uncontrolled cancer proliferation. Although proven chemotherapeutic agents, these compounds target both normal and malignant DNA replication and as such often exhibit deleterious side effects [7–10]. In contrast, few inhibitors have been developed that target replication initiation. As an essential factor that couples DNA replication to both cell cycle progression and checkpoint regulation (below), the Mcm2-7 complex offers a unique and intriguing alternative target for drug development.

Mcm2-7 forms the catalytic core of the helicase (CMG complex, below) that unwinds parental DNA to generate single-stranded templates for DNA polymerase (reviewed in [11]). Mcm2-7 was initially identified during a genetic screen for* S. cerevisiae* mutants that demonstrated defective plasmid segregation (minichromosome maintenance [12]). Subsequent work in yeast demonstrated that such* mcm* alleles cause a replication defect [13], and the corresponding proteins were later found to be components of “licensing factor,” a biochemical activity isolated from* Xenopus* egg extracts that couples cell cycle progression to DNA replication [14]. However, due to the inherent enzymatic and regulatory complexity, the biochemical identification of Mcm2-7 as the replicative helicase took many years of work from multiple laboratories (reviewed in [11]).

**Figure 1:**
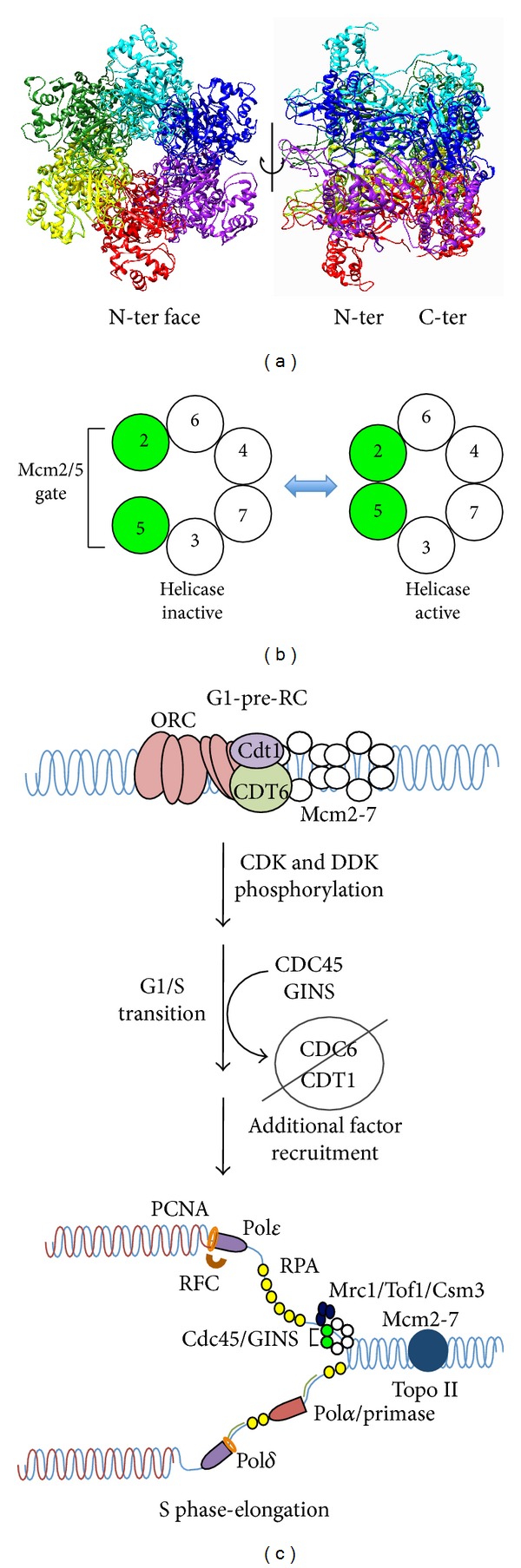
Mcm2-7 is a key regulatory component of cell cycle progression. (a) Homology model of the human Mcm2-7 complex. No high resolution structure yet exists for the eukaryotic Mcm2-7 complex. However, the archaea have homohexameric Mcm helicases, and a crystal structure of the* S. solfataricus* Mcm complex has been solved [58]. To generate a homology model, the human Mcm protein sequences were uploaded into the Phyre 2 server (http://www.sbg.bio.ic.ac.uk/phyre2/) that assigns secondary structure based upon alignment against homologous proteins with solved structures [59]. The resulting Mcm structure predictions were then threaded into an existing hexameric archaeal Mcm structure (PDB ID 2VL6) using PYMOL (http://www.pymol.org) and the previously determined arrangement of adjoining Mcm subunits [18, 19]. As shown, the Mcm2-7 complex generates a toroidal structure resembling the SV-40 large T antigen, a related AAA+ helicase [60]. (b) The Mcm complex is functionally asymmetric. Numerous lines of biochemical and structural evidence demonstrate that the six active sites formed by the six subunits* in trans* are functionally distinct (reviewed in [11]). The Mcm2/5 site has low ATP turnover, suggesting it is regulatory in nature and forms a reversible discontinuity that must be closed in order to activate helicase activity. (c) Mcm2-7 is the key component of S-phase activation (reviewed in [11, 31, 61]). In early G1 phase, Mcm hexamers are recruited to the origin recognition complex (ORC), and bound to origins of replication by the loading factors Cdc6 and Cdt1. The Mcm toroid is bound around dsDNA [35, 62], presumably requiring the ring to be opened at the Mcm 2/5 active site [38]. Along with ORC and Cdc6, head-to-head Mcm2-7 dimers remain in a biochemically inactive state as part of the prereplication complex until their irreversible activation by the regulatory kinases DDK (Dbf4 dependent kinase) and CDK (cyclin dependent kinase). CDC45 and GINS are targeted to the Mcm2-7 complex by the activity of additional recruitment factors such as Sld2, Sld3, and Dbp11, and the Mcm complex shifts from dsDNA bound state to a ssDNA bound state. DNA unwinding commences to provide a ssDNA template for the rest of the DNA replication machinery. Concurrently, Cdc6 and Cdt1 are removed from the nucleus to prevent reloading of the helicase and deleterious rereplication of the genome.

Mcm2-7 is an unusually complex helicase. Unlike prokaryotic and viral hexameric helicases formed from six copies of an identical protein, Mcm2-7 consists of six different subunits (historically numbered from 2 → 7). Although each is distinct and essential [13, 15, 16], these subunits are all AAA+ ATPases and demonstrate partial sequence homology with one another [17]. As is common among AAA+ ATPases, Mcm2-7 forms a toroidal complex with ATPase active sites at dimer interfaces formed from conserved motifs contributed by each adjoining subunit [18, 19] (Figure 1(a)). The six Mcm subunits demonstrate particularly high evolutionary conservation relative to other replication proteins; each subunit defines a gene family that is found in essentially all eukaryotes studied to date [20, 21]. Although most of the structural and mechanistic work to date has been performed on the Mcm2-7 complex from yeast and* Drosophila*, the strong evolutionary conservation of Mcm2-7 makes it likely that findings with lower eukaryotes will also apply to human DNA replication.

The structural complexity of Mcm2-7 appears to be related to its regulation. Both genetic and biochemical investigations demonstrate an unequal functional contribution among these six active sites for DNA unwinding [16, 19, 22–27] (Figure 1(b)). DNA unwinding appears to require only the Mcm4, 6, and 7 subunits, as this particular trimeric subassembly from a variety of different organisms is competent to unwind DNA* in vitro* [26, 28, 29]. Moreover, work from budding yeast has shown that a complex containing only the Mcm4 and 7 subunits is specifically capable of unwinding DNA [25], and biochemical analysis of the corresponding Mcm4/7 ATPase active site demonstrates that it is particularly important for both steady-state ATP hydrolysis and DNA unwinding activities of the Mcm2-7 hexamer [19, 23, 24]. In contrast, the Mcm2/5 ATPase active site serves to regulate the DNA unwinding activity through formation of a reversible discontinuity within the Mcm toroid structure (Mcm2/5 gate, Figure 1(b)): the gate-open conformation blocks helicase activity, whereas the gate-closed conformation is helicase-active [23, 30]. In general, regulation of the Mcm2/5 gate conformation may be the main function of the Mcm2, 3, and 5 subunits, as ablation of the Mcm2/5 ATPase site [22] as well as those flanking the gate (Mcm6/2 and Mcm5/3 [24]) biochemically reduce the ability of Mcm2-7 to alternate between the gate-open and gate-closed forms.

Accumulating evidence indicates that regulation of the Mcm2/5 gate conformation restricts DNA replication to S-phase and ensures that one and only one copy of the genome is replicated per cell cycle. This regulation is a two-step process that involves the Mcm2/5 gate; Mcm2-7 loads onto chromosomes during G1 but is activated for DNA unwinding only following passage into S-phase (Figure 1(c), legend [31]). During initiation in G1, Mcm2-7 origin loading requires several factors (e.g., Cdt1 and Cdc6) which together with the origin recognition complex (ORC) form the prereplication complex (pre-RC [32–35]). Mcm2-7 origin association does not occur passively;* in vitro*, Mcm ATP hydrolysis is required for pre-RC formation [36, 37]. At least one role of this ATP hydrolysis may be Mcm2/5 gate opening, as conditional forced dimerization of the Mcm2 and 5 subunits using Mcm alleles that contain rapamycin-mediated dimerization domains (to mimic the gate closed form) blocks Mcm2-7 DNA loading and cell cycle progression* in vivo* [38]. This effect is specific for the Mcm2 and 5 dimer interface, as forced dimerization between other neighboring subunits has no effect [38].

Structural evidence indicates that closure of the Mcm2/5 gate is required to activate DNA unwinding and elongation. Upon S-phase entry, several regulatory kinases (including the cyclin-dependent kinases (CDKs) and the Dbf4-dependent kinase (DDK)) activate Mcm2-7 by enabling the loading of the key accessary factors Cdc45 and GINS that in combination with Mcm2-7 form the CMG complex (Cdc45-Mcm2-7-GINS) [39–41]. Participation of both Cdc45 and GINS in the CMG complex greatly stimulates the* in vitro *DNA unwinding activity of Mcm2-7, and the* in vivo *formation of the CMG is presumably the main S-phase activation step of DNA replication [27]. Structural analysis of the CMG complex by transmission electron microscopy has determined the mechanism of Cdc45 and GINS activation; together these proteins bind across the Mcm2/5 gate and close the discontinuity [30]. Since Mcm loading and activation are mutually exclusive events (reviewed in [31]), the cell cycle regulation of DNA replication fundamentally centers on the loading and subsequent activation of Mcm2-7.

There is mounting evidence that Mcm2-7 is also a focus of regulation during elongation. The DNA replication checkpoint (DRC) monitors chromosome replication during S-phase; if damage is detected, it promotes genome stability by shutting down cell cycle progression and elongation until the problem is repaired (reviewed in [42]). In the event that the damage is not repaired, the pathway in metazoans eventually causes apoptosis and the elimination of potentially carcinogenic cells from the population [43]. Key to this checkpoint is the ATR sensor kinase and Chk1/2 effector kinases; all, if mutated, promote genome instability leading to cancer [44]. Although the mechanism is yet unclear, circumstantial evidence suggests that Mcm2-7 is regulated by the replication checkpoint. Mcm2-7 is directly phosphorylated during replication stress by ATR [45–48]. Moreover, Mcm2-7 physically associates with three conserved proteins that serve as mediator factors of the DRC (Claspin, Tipin, and Tof1 in metazoans, or Mrc1, Csm3, and Tof1 in budding yeast [49–52]), making it likely that this constitutive association with Mcm2-7 has regulatory significance.

Given the likely differential involvement of specific Mcm2-7 ATPase active sites in multiple aspects of DNA replication and its regulation, small molecule inhibitors could be profitably identified that selectively target these individual activities. Such inhibitors could prove useful for a variety of research as well as chemotherapy applications. Although various* in vitro *DNA replication systems have been established [53–56], dissecting-out the precise mechanistic roles of the various component proteins is difficult, an issue compounded by the fact that many replication factors are ATPases that are difficult to individually inactivate using available nonspecific ATPase inhibitors. Moreover, as various alterations in Mcm expression or function are linked to oncogenic DNA replication (e.g., [57]), Mcm2-7 is a promising drug target for the development of both general replication inhibitors that stem cellular proliferation, as well as potentially more sophisticated inhibitors that specifically target Mcm2-7 in tumor cells (discussed below).

## 2. The Mcms and Cancer

Genomic instability, often caused by replication stress [65], is believed to be a necessary step in cancer development. As such, Mcm2-7 expression levels and activity need to be carefully balanced to preserve genome stability. Although yeast does not develop cancer* per se,* much of our knowledge of how Mcms affect genomic stability stem from studies of these organisms. In addition to the plasmid loss phenotype described earlier [12], Mcm mutations cause chromosome loss, DNA damage, and increased recombination in budding yeast [13, 66]. In* S. pombe*, Mcm mutants have been shown to accumulate DNA repair foci diagnostic of DNA double strand breaks (DSBs) [67]. Moreover, although the number of individual Mcm subunits in the nucleus considerably exceeds the number of replication origins [68, 69], as little as a twofold reduction in Mcm expression has been shown to cause genomic instability [68, 70]. In total, these defects have largely been interpreted as underreplication caused by reduced Mcm2-7 activity [71]. As both DNA replication and fundamental issues of genomic instability are highly conserved among eukaryotes, our knowledge of Mcm2-7 derived from simpler eukaryotes is likely directly relevant to cancer development in metazoan systems.

Consistent with their essential role in cellular proliferation, the Mcms have found common use as a cytological marker of cancer. Since Mcm protein is absent from chromatin in quiescent cells but abundant in active mitotic cells [72], many groups have studied the potential for using Mcm2-7 expression as an immunocytological marker for cellular proliferation [73–75]. Further studies validate the Mcm proteins (Mcm2 in particular) as excellent prognostic and diagnostic markers of human oral, colon, ovarian, and urothelial carcinomas that compare favorably with more traditional cytological markers such as PCNA and Ki-67 (reviewed in [76]).

Studies in both mice and human cells indicate that both* MCM* gene duplication and overexpression can contribute to cancer development (e.g., [77–79]). The recent high-throughput sequencing of various cancerous tissues indicates that the amplification of at least one of the* MCM* genes is relatively common. For example, in a study of 178 tumor genomes that had been corrected for somatic variations, 10% of lung squamous cell carcinomas contained amplifications in at least one* MCM* gene (http://www.cbioportal.org [80]). Moreover, direct reconstruction studies indicate that overexpression of individual Mcm subunits can stimulate cancer formation. Targeted overexpression of* MCM7* in epidermal tissue predisposed mice to form malignant tumors, as animals that overexpressed* MCM7* saw a decrease in the average time to develop tumors in response to carcinogens and an increase in the frequency and propensity of these tumors to form squamous cell carcinomas relative to wild type littermates [81].

Reductions in Mcm2-7 expression levels have also been linked to cancer. Systematic ablation of one of the two gene copies of either Mcm2, 3, 4, or 6, as well as combinations of these hemizygous alleles, have been studied in mice. In general, such mice show reduced MCM proteins levels, growth retardation, and reduced proliferation. Thus, as in budding yeast, MCM protein levels need to be critically managed in metazoans to ensure normal growth. Consistent with genomic instability studies in yeast [68, 70], an experimental reduction of Mcm2 expression in transgenic mice causes lymphomas [82, 83]. Such mice died in early adulthood from various cancers, and necropsy revealed a 100% penetrance of thymomas [83].

Moreover,* MCM* point mutations are common in tumors. For example, in a study of 178 tumor genomes that had been corrected for somatic variations, 12% of lung squamous cell carcinomas were found to contain point mutations in at least one of the six* MCM* genes (http://www.cbioportal.org [80]). Although several* MCM* point mutations have been shown to cause cancer, it is unclear if this is due to a general hypomorphic reduction in DNA replication potential, or a specific loss of Mcm regulation. For example, a specific viable* MCM* allele*, mcm4* ^*chaos3 *^(*mcm4* ^*F345I*^), was identified in a forward genetic screen for cancer-causing mouse alleles and results in spontaneous mammary tumors in 80% of mice [84, 85]. When this same allele was reconstructed into the yeast* MCM4* gene, the corresponding* S. cerevisiae* mutant demonstrated a classical plasmid loss phenotype, genomic instability, and reduced viability [84, 86]. In this case, the* mcm* ^*chaos3*^ allele was shown to generate Mcm2-7 complexes with reduced physical stability, suggesting that the* chaos3* allele functions to nonspecifically reduce DNA replication potential [85]. In contrast, a second* mcm4* allele was identified as a spontaneous dominant mutation in a mouse colony that had acquired an early-onset leukemogenesis phenotype. The cancer phenotype was subsequently mapped to* MCM4*, and the relevant amino acid substitution (*mcm*4^D573H^) was found to occur in the universally conserved Walker B ATPase motif. Unlike* mcm4* ^*chaos3*^, the mutant was not hypomorphic and failed to compliment a* MCM4* deletion in yeast complementation assays, suggesting that *mcm*4^D573H^ is a dominant change of function allele that poisons normal Mcm2-7 helicase activity [87].

In total these mouse and tissue culture studies strongly imply that Mcm alterations can also drive human cancer. In normal human genomes, various single nucleotide polymorphisms in the* mcm* genes are commonly observed (http://www.ncbi.nlm.nih.gov/projects/SNP/). At least some of these polymorphisms may in themselves generate genomic instability in susceptible individuals, as at least some MCM polymorphisms cause genomic instability when assayed in budding yeast [88]. Intriguingly, among* mcm* cancer alleles listed in the cBioPortal (http://www.cbioportal.org), mutations that fall within the conserved ATPase motifs (Walker A and B, Sensor 1 and 2, and Arginine finger motif) commonly occur among all six Mcm genes. As such, some of these alleles may generate Mcm2-7 complexes with a specific biochemical defect in a particular step of DNA replication or its regulation rather than generating a generally hypomorphic situation.

Thus, the role of the Mcms in cancer development seems contradictory, as both underexpression (consistent with a tumor suppressor) and overexpression (consistent with an oncogene) are linked to cancer development. Although a direct Mcm2-7-mediated biochemical defect in DNA replication cannot be ruled out in either case, the underlying causes behind these two conditions are likely to be very different, while. Underexpression likely reduces the level of Mcm2-7 complexes needed for normal DNA replication, while overexpression likely reflects inappropriate protein-protein interactions. Such interactions might serve to either titrate out factors that block abnormal proliferation (e.g., Rb, below), or upset a critical stoichiometric balance among Mcm subunits within the cell to increase nonproductive Mcm subassemblies at the expense of active hexamers. Alternatively, Mcm gene overexpression may lead to higher concentrations functional Mcm2-7 complexes per cell, resulting in a deleterious increase of origin activation and/or DNA unwinding. However, under either scenario, excess Mcm2-7 activity either directly or indirectly drives cellular proliferation. In total, these studies collectively provide strong evidence for a functional connection between the Mcm complex and cancer development, and modulating their activity may be an avenue for the development of novel therapeutics.

## 3. Various Tumor Suppressors and Regulatory Factors Bind Mcm2-7 and Inhibit Its Activity

Accumulating evidence suggests that during early cancer development, altered Mcm2-7 regulation resulting from oncogene expression leads to a particularly mutagenic form of DNA replication (oncogene-induced DNA replication stress [89–92]) that fuels genomic instability and proliferation. Evidence derived from the sequencing of tumor genomes suggests that such oncogenic replication stress occurs through alterations in Rb/E2F regulation and the control of G1/S phase progression, resulting in the production of DNA double-stranded breaks (DSBs), genomic instability and mutagenesis, and the subsequent loss of key regulators such as the p53 tumor suppressor (reviewed in [89, 90]).

**Figure 2:**
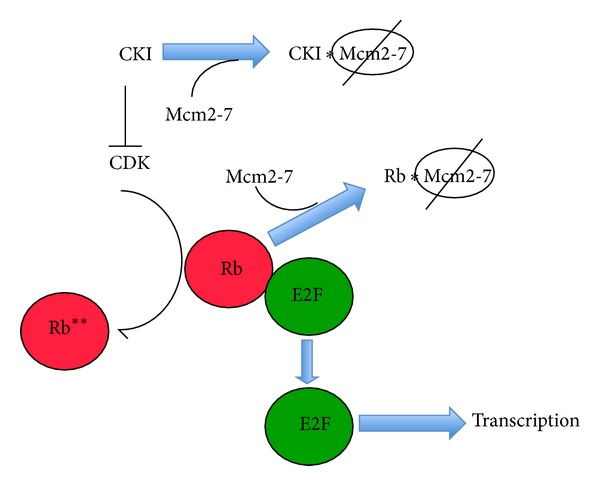
A simplified overview of the Rb/E2F pathway. In general, Rb binds to and inhibits E2F, resulting in the altered transcription of numerous S-phase relevant genes. During the G1/S part of the cell cycle, increased CDK activity leads to RB phosphorylation, which causes release and activation of E2F, and an induction of S-phase dependent gene expression. In turn, various inhibitors (CKI) modulate CDK activity. In addition to their well-established role in transcriptional regulation through E2F, both CKIs and Rb bind to and inactivate Mcm2-7; how this inhibition is reverded to facilitate subsequent DNA replication is currently unknown.

The Rb (retinoblastoma) protein family members normally inhibit S-phase progression by binding to and subsequently inactivating members of the E2F family (reviewed in [93], Figure 2). The Rb family contains related factors with somewhat different properties; these include p105, p107, and p130 proteins [93]. In contrast the E2F proteins are transcriptional activators or repressors that directly control the transition into S-phase by modulating gene expression. Progression into S-phase depends upon CDK activity; Rb phosphorylation by CDK promotes E2F release and activates its transcription function. In turn, the CDKs themselves are inhibited by various regulatory proteins (e.g., CDK inhibitors (CKI)). Multiple CKIs exist in cells, and among others form the INK4 (e.g., p16^INK4a^, p15^INK4b^, p18^NK4c^, and p19^INK4d^) and KIP/CIP (e.g., p27^KIP1^ and p21^CIP1^) families [94, 95]. As such, both Rb and CKIs are inhibitors of cell cycle progression, and members of both families are commonly mutated in human tumors [96].

Although the details are yet unclear, altered replication origin firing may be the underlying cause behind oncogene-induced replication stress [65]. Work done in both yeast as well as metazoans suggests the existence of an optimal level of origin usage: both too few and too many firing origins lead to DSB formation (reviewed in [65]). As discussed above, the Mcms are the fundamental focus of both origin loading and activation, raising the strong likelihood that Mcm misregulation plays a role in oncogene-induced replication stress.

In support of this conjecture, both Rb and several CKIs have been shown to bind Mcm7 and inhibit Mcm2-7 activity (Table 1, Figure 2). A yeast two-hybrid screen aimed at identifying proteins that bind the N-terminal region of Rb showed that it forms a complex with the carboxy terminus of Mcm7. Immunoprecipitations with full length Rb (p105) and Mcm7 proteins recapitulated this interaction* in vitro *and also demonstrated that other Rb family members p107 and p130 also bind Mcm7 [97]. Furthermore, Rb and p130 inhibited DNA replication in a* Xenopus *DNA replication assay in an Mcm7-dependent manner [98], suggesting that physical interactions between Mcm7 and Rb have physiological significance.

Several CKIs have also been found to block Mcm2-7 function (Figure 2). The cyclin-D dependent kinase inhibitor p16^INK4a  ^ has been shown to indirectly block Mcm origin loading by inhibiting the activities of Cdc6 and Cdt1 [101]. In contrast, the p27^kip1^ factor, a CDK and DNA replication inhibitor (reviewed in [105]) has been shown to bind the AAA+ motor domain of Mcm7 [102]. This interaction appears physiologically relevant, as a truncated p27^kip1^ protein capable of binding Mcm7 but lacking the ability to inhibit CDK was able to cause significant inhibition of DNA replication in an* in vitro* DNA replication system [102]. This implies that the Mcm7/p27^kip1^ interaction can regulate DNA replication independent of CDK inhibition.

In addition to factors directly involved in Rb/E2F regulation, other regulatory factors bind Mcm2-7 in an apparently functional manner. (1) Prohibitin, a scaffolding protein that, similar to Rb, had previously been shown to inhibit E2F transcription targets [106, 107], was found to physically interact primarily with Mcm2 and 5, perhaps functioning by interfering with the Mcm2/5 gate. Purified prohibitin also inhibited* in vitro* DNA replication, perhaps by inhibiting Mcm2-7 [103]. (2) The human Rad17 protein, which together with RFC 2-5 forms an alternative clamp loader that (along with the 911 complex) is required for ATR activation of the replication checkpoint cascade in metazoans [108], binds the C-terminus of Mcm7 [104]. Transfection of just the Mcm7-binding region of Rad17 into cell lines abolished UV-induced replication checkpoint activation, suggesting that this interaction is physiologically relevant [104]. (3) Recent work has demonstrated that the NCOA4 transcriptional coactivator also binds Mcm7 in a relevant manner to inhibit DNA replication by interacting with the CMG complex, blocking its helicase activity, and negatively regulating the activation of origins of replication [100]. (4) Finally, a variety of additional proteins have been shown to bind the C-terminus of Mcm7, but currently the physiological significance of the observed binding interactions is unknown or poorly understood. These include the ING5 tumor suppressor [99], ATRIP [45], and Cyclin D1 dependent kinase [109].

Although the mapping of specific interaction sites between Mcm7 and its various binding partners has not yet been performed at high resolution, available evidence suggests that these sites likely overlap conserved ATPase motifs. Rb binds a region of Mcm7 that is contained within a fragment encoding amino acids 583–719 of human Mcm7 [97], while Rad17 binding is contained within amino acids 521–620 [104]. Both putative interaction sites span the conserved Sensor 2 and Presensor 2 motifs of Mcm7 that together form part of the Mcm4/7 ATPase active site [11]. As this region spans essential active site motifs, it is evolutionary well conserved particularly among metazoans, and the binding of these regulatory factors to Mcm7 likely functions to block or alter ATP hydrolysis at the Mcm4/7 site.

Thus, the observed interactions between Mcm2-7 and the various regulatory factors may target key enzymatic activities, either DNA unwinding or regulation of the Mcm2/5 gate. The connection between Mcm2-7 and multiple members of the Rb/E2F signaling pathway appears to be direct and distinct from the role of this pathway in modulating gene expression. Finally, as most reported Mcm7 binding interactions target the Mcm7 C-terminus, competition among these factors for Mcm7 binding may be an important aspect of Mcm2-7 regulation.

## 4. Small Molecule Inhibitors and Potential Chemotherapeutic Agents of the Eukaryotic Replicative Helicase 

Helicases are common enzymes. For example,* S. cerevisiae* contains 134 open reading frames (2% of its genome) that encode proteins containing helicase structural motifs [110]. Helicases in general have received recent drug-discovery attention, and small molecule inhibitors of viral helicases have been the focus of several high throughput screens (reviewed in [111]). Many viral helicases (e.g., SV40 large T antigen [112]) have multiple cellular functions in addition to bulk replication. This property increases the potency of such small molecule inhibitors, as more cellular systems are coordinately impacted, and the likelihood of acquiring drug resistance mutations is decreased. Mcm2-7 is similar in that it coordinates regulatory processes in addition to genome replication. Moreover, the heterohexameric organization of Mcm2-7 might prove particularly advantageous; it might be difficult for an organism to develop drug resistance if multiple Mcm ATPase active sites are targeted. However, in contrast to their prokaryotic and viral counterparts, no high throughput biochemical screens have been performed on the eukaryotic replicative helicase. The reason for this is largely practical: it is difficult to purify Mcm2-7 or the CMG complex in amounts large enough to perform these screens, and* in vitro* helicase activity has not been demonstrable for the whole complex until fairly recently [23, 113].

Given what is known about the biochemistry and genetics of Mcm2-7, one can broadly envision at least three different classes of small molecule inhibitors with potential chemotherapeutic utility. These include (1) enzymatic inhibitors (e.g., targeting the various ATPase active sites), (2) inhibitors that block physical or genetic interactions between Mcm subunits and other proteins, and (3) molecules that modulate Mcm expression levels.

Enzymatic inhibitors that block Mcm2-7's normal role in either DNA replication initiation or elongation are one obvious class. As mentioned in the Introduction, many types of inhibitors have been developed to block the function of specific replication factors as a means to block the cellular proliferation observed in cancer. Such inhibitors that target Mcm2-7 would potentially provide an additional useful weapon in this arsenal.

Two problems however exist with the identification of therapeutically useful biochemical inhibitors. First, as mentioned above, both the Mcm2-7 and CMG complexes are difficult to purify in sufficient quantity for extensive primary high-throughput screening. Although improved technology may ultimately solve this problem, cell-based screening approaches using engineered test organisms might be devised to identify Mcm inhibitors in a primary screen; such drug candidates could then be subsequently tested in appropriate secondary biochemical screens (e.g., [114]). Second, reduction of Mcm levels as little as twofold below endogenous levels has been shown to cause genomic instability, suggesting that a loss of Mcm activity is deleterious to healthy cells. However, it should be noted that many current chemotherapeutic agents induce genomic instability either as collateral damage (e.g., [115]), or to intentionally trigger apoptosis in sensitive (e.g., cancerous) cells (reviewed in [116]). Moreover, it should be noted that most of the genomic instability defects demonstrated by Mcm mutations are likely the results of elongation problems (e.g., replication fork collapse); potential Mcm inhibitors that block initiation (and hence formation of the replication fork) would likely block this form of genomic instability. In short, it may be possible to develop appropriate inhibitors for Mcm2-7 that balance chemotherapeutic utility with potential off-target genome instability effects.

Alternatively, targeted inhibitors that disrupt interaction between Mcm2-7 and other cellular proteins may be identified that specifically block abnormal DNA replication. Proteins that functionally interact within a cell often demonstrate a property termed synthetic lethality; mutations in either gene may individually support viability, but when combined caused lethality [117]. Thus, inhibitors of Mcm2-7 interacting proteins might be obtained that specifically target abnormal replication caused by Mcm mutants, while having little effect on cells with normal DNA replication. High throughput inhibitor screens that utilize synthetic lethality as a read-out have been developed (reviewed in [118]); the recent identification of PARP inhibitors that specifically target mutant BRAC1-containing cancer cells are an example of such a successful screening approach (reviewed in [119]).

Finally, chemotherapeutics might be identified to specifically tailor Mcm gene expression levels. Since Mcm-2-7 levels appears to be critically balanced to prevent genome instability, drugs that modulate Mcm2-7 gene expression could be profitably developed to either block cellular proliferation or potentially return it to normal levels. Alternatively, under conditions of replication stress (as is the case in cancer cells), specific reduction of Mcm protein levels sensitizes cells to other replication inhibitors [120], suggesting that combinational therapy with Mcm-specific inhibitors has the potential to increase the efficacy of existing treatments and their specificity for cancer cells. Although development of an inhibitor that specifically targets expression of very limited set of genes seems daunting, several recently discovered Mcm inhibitors show promise in this area (e.g., trichostatin A and widdrol).

To date, only a few compounds have been identified using low throughput or candidate approaches that directly target the complex's enzymatic activity and/or expression (Table 2).

(1) Heliquinomycin was originally identified as an inhibitor of* in vitro* replication in cell extract systems [126] and was later shown to biochemically inhibit the DNA unwinding properties of a specific Mcm subcomplex (Mcm467). This inhibition may be indirect, as it is believed that heliquinomycin blocks unwinding via an interaction with single-stranded DNA [121]. The drug may also have* in vivo* utility against Mcm2-7 as it has been shown to selectively decrease the proliferation of cancer cells overexpressing Mcm7 in tissue culture [122].

(2) A recent study has found that the fluoroquinolone ciprofloxacin and related compounds are able to selectively inhibit Mcm2-7 helicase activity at ~3–8-fold lower concentrations relative to other helicases [63]. Although the IC_50_ of this inhibition was relatively weak (~600 *μ*M), cytotoxicity assays demonstrated that ciprofloxacin was able to inhibit both yeast and human cells at concentrations comparable to those that block* in vitro* helicase activity, consistent with the possibility that Mcm2-7 was also a cellular target of ciprofloxacin. This supposition was further supported by the finding that a known* Mcm* yeast mutant (*mcm4* ^chaos*3*^) demonstrated significant ciprofloxacin resistance in cellular culture.

(3) The classical histone deacetylase inhibitor trichostatin A (TSA) has been the subject considerable interest as an anticancer compound and has been demonstrated to be effective against a wide variety of cancers [127]. Recent evidence suggests that* MCM2* is a target of TSA. RT-PCR showed that* MCM2* gene expression is downregulated upon TSA treatment and that knockdown of Mcm2 induces cellular apoptosis in colon cancer cells. This downregulation of* MCM2* was dependent on TSA-mediated changes in the JNK signaling pathway [123].

(4) Widdrol, a naturally occurring aromatic compound derived from* Juniperus chinensis*, was observed to have antiproliferative activity against human colon adenocarcinoma HT29 cells [124]. Interestingly, this effect appeared to be due to a downregulation of* MCM* gene expression as a downstream consequence of DNA damage. The compound was later shown to cause DSBs which activate the DNA damage ATM/ATR mediated checkpoint, resulting in an upregulation of p21^CIP1^ and a rapid decrease of MCM4 levels in HT29 cells, but not mouse fibroblasts [125]. Although the authors proposed that widdrol directly causes DNA damage, this DNA damage phenotype may occur through involvement of the Mcm complex, as both an* mcm* mutation in* S. pombe* has been shown to cause DSBs [67] and recently Mcm4 has been implicated in the DNA damage checkpoint [128].

## 5. Prospects for Mcm2-7 Chemotherapeutics

The development of Mcm2-7-specific small molecule inhibitors is at an early stage, and structure-activity relationship of these compounds is poorly understood. To date, the best information on potential molecular scaffolds for Mcm2-7 inhibitors comes from a study of Mcm2-7 fluoroquinolone inhibitors, as well as inhibitor studies targeting two related AAA+ ATPases—SV40 large T antigen and p97.

**Figure 3:**
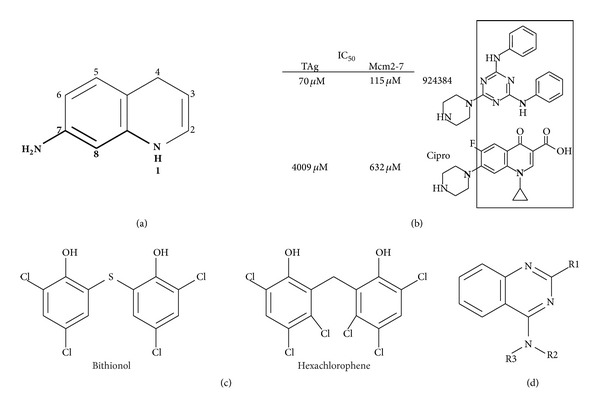
AAA+inhibitors. (a) The basic quinolone structure and substitution numbering scheme are shown. From a previous study [63], most of the better Mcm2-7 inhibitors required both the nitrogen at position one and a nitrogen-containing substituent at position 7, with variation to the position 7 substituent often appearing to strongly module inhibitor activity. (b) Examination of inhibition of Mcm2-7 and SV-40 large T antigen comparing related fluoroquinolone and triazole inhibitors [63]. (c) SV-40 large T antigen inhibitors shown [64]. (d) Basic quinazoline structure shown; R-group substituents are discussed in the text.

*(1) Fluoroquinolones*. To find better inhibitors than ciprofloxacin and elucidate the structural activity relationship between Mcm2-7 and fluoroquinolones, a library of ~150 additional compounds containing various fluoroquinolone substructures was tested biochemically for Mcm2-7-mediated DNA unwinding [63]. Although no inhibitors of greater specificity than ciprofloxacin were identified (i.e., increased ability to discriminate between SV-40 T antigen and Mcm2-7), inhibitors of greater potency were obtained, and common conserved features among them were evident. Particularly key appears to be the nature of a nitrogen-containing substituent arising from the quinolone 7 position, as well as the nitrogen present at the N1 position (Figure 3(a)). These structural features extend to various triazole compounds used in this study that also inhibit Mcm2-7 (e.g., 924384, Figure 3(b)). Since fluoroquinolones have a long history of use as antibiotics, development of Mcm2-7 fluoroquinolone-based inhibitors is attractive as much of the relevant pharmacology of this scaffold has been well-studied [129]. 

*(2) Bisphenols.* T antigen is a AAA+ ATPase and hexameric replicative helicase needed for SV-40 viral DNA replication (reviewed in [130]). A recent high-throughput screen for SV40 Large T antigen (TAg) inhibitors utilized the Spectrum Collection library of ~2200 FDA-approved bioactive compounds. This screen identified bisphenols as a novel compound class that inhibits both the ATPase activity of TAg* in vitro* and the ability of SV-40 to replicate* in vivo* [131]. Two particular molecules (bithionol and hexachlorophene, Figure 3(c)) were discovered with an IC_50_ for TAg ATPase activity in the single micromolar range. Further analysis determined that the critical structural components for TAg inhibition were flexibility at the linker between the phenol groups and the presence of small substituents at positions 2 and 4 of the phenols [131]. 

*(3) Quinazolines*. The p97/CDC48 ATPase is another toroidal eukaryotic hexameric AAA+ protein that in contrast to Mcm2-7 uses ATP-dependent conformation changes to unfold proteins (reviewed in [132]). Quinazoline derivatives have been shown to be effective p97 inhibitors [133]. Toward development of better inhibitors, a structure-activity study tested an additional 200 quinazoline analogs for p97 inhibition, resulting in discovery of two new inhibitors (ML240 and ML241) that each had IC_50_s for p97 ATP hydrolysis at submicromolar concentrations [134]. Their results indicate that substituent alterations at the R1 position greatly modulate p97 inhibition, a benzyl group at R2 is preferred, and substitution of the R3 hydrogen with anything larger blocks p97 inhibition (Figure 3(d)). Quinazoline derivatives may prove to be a generally useful inhibitor scaffold, as an independent study has found that a quinazoline-like compound (ciliobrevin) inhibits the ATPase activity of the AAA+ dynein motor protein [135].

## 6. Conclusions

Mcm2-7 is a structurally and functionally complex replication factor with a rich binding surface that directs multiple regulatory interactions of cancer significance, including those required for both Rb/E2F signaling as well as DNA replication. Given that all of these processes in isolation have been studied or used as therapeutic targets, Mcm2-7's involvement with all three suggests it is a promising target for blocking the proliferation of cancerous and precancerous cells. As Mcm2-7 contains six unique ATPase active sites and binds numerous regulatory proteins to a variety of different sites within the complex, inhibitors could be targeted to disrupt specific regulatory interactions. Although the ability to perform high throughput biochemical screens to identify Mcm2-7 inhibitors has limited utility due to the complexity of this system, recent developments using carefully engineered test organisms and whole cell assays, perhaps in conjunction with simpler and more genetically tractable model systems, suggest ways to conduct such targeted Mcm2-7 screens to identify novel inhibitors with therapeutic potential [114, 118, 136, 137].

## Figures and Tables

**Table 1:** Protein interactors and regulators of the Mcm2*-*7 complex.

Inhibitor	Phenotype	Subunits targeted	Reference
ING5	Binds Mcm2*-*7	Mcm2, 4, 6, & 7	[99]
NCOA4	Blocks origin firing, helicase activity	Mcm7	[100]
p16^INK4a^	Blocks Mcm2*-*7 origin loading	Indirect (Cdc6, Cdt1)	[101]
p27^KIP1^	Blocks *in vitro* replication	Mcm7	[102]
Prohibitin	Blocks *in vitro* replication	Mcm2 & 5	[103]
RAD17	Blocks checkpoint activation	Mcm7	[104]
Retinoblastoma protein/p130	Blocks *in vitro* replication	Mcm7	[97]

**Table 2:** Small molecule inhibitors of Mcm2*-*7.

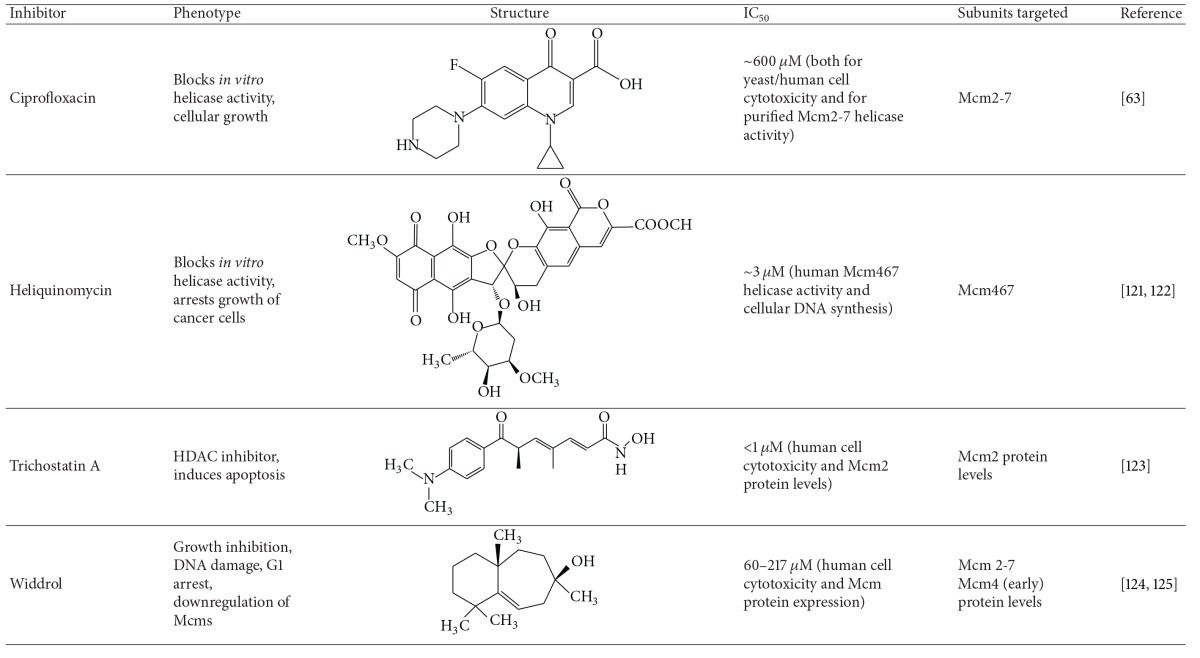

## References

[1] Pogorelčnik B, Perdih A, Solmajer T (2013). Recent developments of DNA poisons - human DNA topoisomerase IIα inhibitors—as anticancer agents. *Current Pharmaceutical Design*.

[2] Keating MJ, Kantarjian H, Talpaz M (1989). Fludarabine: a new agent with major activity against chronic lymphocytic leukemia. *Blood*.

[3] Singh DK, Krishna S, Chandra S, Shameem M, Deshmukh AL, Banerjee D (2014). Human DNA ligases: a comprehensive new look for cancer therapy. *Medicinal Research Reviews*.

[4] Tan Z, Wortman M, Dillehay KL (2012). Small-molecule targeting of proliferating cell nuclear antigen chromatin association inhibits tumor cell growth. *Molecular Pharmacology*.

[5] Aye Y, Li M, Long MJC, Weiss RS (2014). Ribonucleotide reductase and cancer: biological mechanisms and targeted therapies. *Oncogene*.

[6] Maji B, Bhattacharya S (2014). Advances in the molecular design of potential anticancer agents via targeting of human telomeric DNA. *Chemical Communications*.

[7] Anderson VR, Perry CM (2007). Fludarabine: a review of its use in non-Hodgkin's lymphoma. *Drugs*.

[8] Chun HG, Leyland-Jones BR, Caryk SM, Hoth DF (1986). Central nervous system toxicity of fludarabine phosphate. *Cancer Treatment Reports*.

[9] Mazevet M, Moulin M, Llach-Martinez A (2013). Complications of chemotherapy, a basic science update. *La Presse Médicale*.

[10] Livshits Z, Rao RB, Smith SW (2014). An approach to chemotherapy-associated toxicity. *Emergency Medicine Clinics of North America*.

[11] Bochman ML, Schwacha A (2009). The Mcm complex: unwinding the mechanism of a replicative helicase. *Microbiology and Molecular Biology Reviews*.

[12] Maine GT, Sinha P, Tye BW (1984). Mutants of S. cerevisiae defective in the maintenance of minichromosomes. *Genetics*.

[13] Hennessy KM, Lee A, Chen E, Botstein D (1991). A group of interacting yeast DNA replication genes. *Genes and Development*.

[14] Thömmes P, Kubota Y, Takisawa H, Julian Blow J (1997). The RLF-M component of the replication licensing system forms complexes containing all six MCM/P1 polypeptides. *The EMBO Journal*.

[15] Labib K, Tercero JA, Diffley JFX (2000). Uninterrupted MCH2-7 function required for DNA replication fork progression. *Science*.

[16] Schwacha A, Bell SP (2001). Interactions between two catalytically distinct MCM subgroups are essential for coordinated ATP hydrolysis and DNA replication. *Molecular Cell*.

[17] Koonin EV (1993). A common set of conserved motifs in a vast variety of putative nucleic acid-dependent ATPases including MCM proteins involved in the initiation of eukaryotic DNA replication. *Nucleic Acids Research*.

[18] Davey MJ, Indiani C, O'Donnell M (2003). Reconstitution of the Mcm2-7p heterohexamer, subunit arrangement, and ATP site architecture. *Journal of Biological Chemistry*.

[19] Bochman ML, Bell SP, Schwacha A (2008). Subunit organization of Mcm2-7 and the unequal role of active sites in ATP hydrolysis and viability. *Molecular and Cellular Biology*.

[20] Iyer LM, Aravind L, DePamphilis ML (2006). The evolutionary history of proteins involved in pre-replication complex assembly. *DNA Replication and Human Disease*.

[21] Aves SJ, Liu Y, Richards TA (2012). Evolutionary diversification of eukaryotic DNA replication machinery. *Subcellular Biochemistry*.

[22] Bochman ML, Schwacha A (2007). Differences in the single-stranded DNA binding activities of MCM2-7 and MCM467: MCM2 and MCM5 define a slow ATP-dependent step. *Journal of Biological Chemistry*.

[23] Bochman ML, Schwacha A (2008). The Mcm2-7 complex has in vitro helicase activity. *Molecular Cell*.

[24] Bochman ML, Schwacha A (2010). The Saccharomyces cerevisiae Mcm6/2 and Mcm5/3 ATPase active sites contribute to the function of the putative Mcm2-7 “gate”. *Nucleic Acids Research*.

[25] Kanter DM, Bruck I, Kaplan DL (2008). Mcm subunits can assemble into two different active unwinding complexes. *Journal of Biological Chemistry*.

[26] Ishimi Y (1997). A DNA helicase activity is associated with an MCM4, -6, and -7 protein complex. *Journal of Biological Chemistry*.

[27] Ilves I, Petojevic T, Pesavento JJ, Botchan MR (2010). Activation of the MCM2-7 helicase by association with Cdc45 and GINS proteins. *Molecular Cell*.

[28] Lee J, Hurwitz J (2000). Isolation and characterization of various complexes of the minichromosome maintenance proteins of Schizosaccharomyces pombe. *Journal of Biological Chemistry*.

[29] Kaplan DL, Davey MJ, O'Donnell M (2003). Mcm4,6,7 uses a “pump in ring” mechanism to unwind DNA by steric exclusion and actively translocate along a duplex.. *The Journal of biological chemistry*.

[30] Costa A, Ilves I, Tamberg N (2011). The structural basis for MCM2-7 helicase activation by GINS and Cdc45. *Nature Structural & Molecular Biology*.

[31] Bell SP, Dutta A (2002). DNA replication in eukaryotic cells. *Annual Review of Biochemistry*.

[32] Aparicio OM, Weinstein DM, Bell SP (1997). Components and dynamics of DNA replication complexes in S. cerevisiae: redistribution of MCM proteins and Cdc45p during S phase. *Cell*.

[33] Tanaka T, Knapp D, Kim N (1997). Loading of an Mcm protein onto DNA replication origins is regulated by Cdc6p and CDKs. *Cell*.

[34] Sun J, Evrin C, Samel SA (2013). Cryo-EM structure of a helicase loading intermediate containing ORC-Cdc6-Cdt1-MCM2-7 bound to DNA. *Nature Structural and Molecular Biology*.

[35] Remus D, Beuron F, Tolun G, Griffith JD, Morris EP, Diffley JFX (2009). Concerted loading of Mcm2-7 double hexamers around DNA during DNA replication origin licensing. *Cell*.

[36] Coster G, Frigola J, Beuron F (2014). Origin licensing requires ATP binding and hydrolysis by the MCM replicative helicase. *Molecular Cell*.

[37] Kang S, Warner MD, Bell SP (2014). Multiple functions for Mcm2-7 ATPase motifs during replication initiation. *Molecular Cell*.

[38] Samel SA, Fernández-Cid A, Sun J (2014). A unique DNA entry gate serves for regulated loading of the eukaryotic replicative helicase MCM2-7 onto DNA. *Genes & Development*.

[39] Zegerman P, Diffley JFX (2007). Phosphorylation of Sld2 and Sld3 by cyclin-dependent kinases promotes DNA replication in budding yeast. *Nature*.

[40] Takayama Y, Kamimura Y, Okawa M, Muramatsu S, Sugino A, Araki H (2003). GINS, a novel multiprotein complex required for chromosomal DNA replication in budding yeast. *Genes and Development*.

[41] Tanaka S, Umemori T, Hirai K, Muramatsu S, Kamimura Y, Araki H (2007). CDK-dependent phosphorylation of Sld2 and Sld3 initiates DNA replication in budding yeast. *Nature*.

[42] Branzei D, Foiani M (2006). The Rad53 signal transduction pathway: replication fork stabilization, DNA repair, and adaptation. *Experimental Cell Research*.

[43] Cho Y, Liang P (2011). S-phase-coupled apoptosis in tumor suppression. *Cellular and Molecular Life Sciences*.

[44] Motoyama N, Naka K (2004). DNA damage tumor suppressor genes and genomic instability. *Current Opinion in Genetics and Development*.

[45] Cortez D, Glick G, Elledge SJ (2004). Minichromosome maintenance proteins are direct targets of the ATM and ATR checkpoint kinases. *Proceedings of the National Academy of Sciences of the United States of America*.

[46] Ishimi Y, Komamura-Kohno Y, Kwon H, Yamada K, Nakanishi M (2003). Identification of MCM4 as a target of the DNA replication block checkpoint system. *The Journal of Biological Chemistry*.

[47] Randell JCW, Fan A, Chan C (2010). Mec1 is one of multiple kinases that prime the Mcm2-7 helicase for phosphorylation by Cdc7. *Molecular Cell*.

[48] Yoo HY, Shevchenko A, Dunphy WG (2004). Mcm2 is a direct substrate of ATM and ATR during DNA damage and DNA replication checkpoint responses. *The Journal of Biological Chemistry*.

[49] Bando M, Katou Y, Komata M (2009). Csm3, Tof1, and Mrc1 form a heterotrimeric mediator complex that associates with DNA replication forks. *The Journal of Biological Chemistry*.

[50] Chou DM, Elledge SJ (2006). Tipin and Timeless form a mutually protective complex required for genotoxic stress resistance and checkpoint function. *Proceedings of the National Academy of Sciences of the United States of America*.

[51] Katou Y, Kanoh Y, Bando M (2003). S-phase checkpoint proteins Tof1 and Mrc1 form a stable replication-pausing complex. *Nature*.

[52] Nedelcheva MN, Roguev A, Dolapchiev LB (2005). Uncoupling of unwinding from DNA synthesis implies regulation of MCM helicase by Tof1/Mrc1/Csm3 checkpoint complex. *Journal of Molecular Biology*.

[53] Chong JPJ, Thömmes P, Rowles A, Mahbubani HM, Blow JJ (1997). Characterization of the Xenopus replication licensing system. *Methods in Enzymology*.

[54] Seki T, Diffley JFX (2000). Stepwise assembly of initiation proteins at budding yeast replication origins in vitro. *Proceedings of the National Academy of Sciences of the United States of America*.

[55] Pasero P, Gasser SM (2002). In vitro DNA replication assays in yeast extracts. *Methods in Enzymology*.

[56] Stillman B, Gerard RD, Guggenheimer RA, Gluzman Y (1985). T antigen and template requirements for SV40 DNA replication in vitro. *The EMBO Journal*.

[57] Flach J, Bakker ST, Mohrin M (2014). Replication stress is a potent driver of functional decline in ageing haematopoietic stem cells. *Nature*.

[58] Brewster AS, Wang G, Yu X (2008). Crystal structure of a near-full-length archaeal MCM: functional insights for an AAA+ hexameric helicase. *Proceedings of the National Academy of Sciences of the United States of America*.

[59] Kelley LA, Sternberg MJE (2009). Protein structure prediction on the Web: a case study using the Phyre server.. *Nature protocols*.

[60] Li D, Zhao R, Lilyestrom W (2003). Structure of the replicative helicase of the oncoprotein SV40 large tumour antigen. *Nature*.

[61] Riera A, Tognetti S, Speck C (2014). Helicase loading: how to build a MCM2-7 double-hexamer. *Seminars in Cell and Developmental Biology*.

[62] Evrin C, Clarke P, Zech J (2009). A double-hexameric MCM2-7 complex is loaded onto origin DNA during licensing of eukaryotic DNA replication. *Proceedings of the National Academy of Sciences of the United States of America*.

[63] Simon N, Bochman ML, Seguin S, Brodsky JL, Seibel WL, Schwacha A (2013). Ciprofloxacin is an inhibitor of the Mcm2-7 replicative helicase. *Bioscience Reports*.

[64] Seguin SP, Evans CW, Nebane-Akah M (2012). High-throughput screening identifies a bisphenol inhibitor of SV40 large T antigen ATPase activity. *Journal of Biomolecular Screening*.

[65] Hills SA, Diffley JF (2014). DNA replication and oncogene-induced replicative stress. *Current Biology*.

[66] Gibson SI, Surosky RT, Tye B- (1990). The phenotype of the minichromosome maintenance mutant mcm3 is characteristic of mutants defective in DNA replication. *Molecular and Cellular Biology*.

[67] Bailis JM, Luche DD, Hunter T, Forsburg SL (2008). Minichromosome maintenance proteins interact with checkpoint and recombination proteins to promote S-phase genome stability. *Molecular and Cellular Biology*.

[68] Lei M, Kawasaki Y, Tye BK (1996). Physical interactions among Mcm proteins and effects of Mcm dosage on DNA replication in Saccharomyces cerevisiae. *Molecular and Cellular Biology*.

[69] Woodward AM, Göhler T, Luciani MG (2006). Excess Mcm2-7 license dormant origins of replication that can be used under conditions of replicative stress. *Journal of Cell Biology*.

[70] Liang DT, Hodson JA, Forsburg SL (1999). Reduced dosage of a single fission yeast MCM protein causes genetic instability and S phase delay. *Journal of Cell Science*.

[71] Bailis JM, Forsburg SL (2004). MCM proteins: DNA damage, mutagenesis and repair. *Current Opinion in Genetics and Development*.

[72] Madine MA, Swietlik M, Pelizon C, Romanowski P, Mills AD, Laskey RA (2000). The roles of the MCM, ORC, and Cdc6 proteins in determining the replication competence of chromatin in quiescent cells. *Journal of Structural Biology*.

[73] Freeman A, Morris LS, Mills AD (1999). Minichromosome maintenance proteins as biological markers of dysplasia and malignancy. *Clinical Cancer Research*.

[74] Todorov IT, Werness BA, Wang H (1998). HsMCM2/BM28: a novel proliferation marker for human tumors and normal tissues. *Laboratory Investigation*.

[75] Stoeber K, Tlsty TD, Happerfield L (2001). DNA replication licensing and human cell proliferation. *Journal of Cell Science*.

[76] Giaginis C, Vgenopoulou S, Vielh P, Theocharis S (2010). MCM proteins as diagnostic and prognostic tumor markers in the clinical setting. *Histology and Histopathology*.

[77] Ren B, Yu G, Tseng GC (2006). MCM7 amplification and overexpression are associated with prostate cancer progression. *Oncogene*.

[78] Qian L, Luo Q, Zhao X, Huang J (2014). Pathways enrichment analysis for differentially expressed genes in squamous lung cancer. *Pathology Oncology Research*.

[79] Chuang C, Wallace MD, Abratte C, Southard T, Schimenti JC (2010). Incremental genetic perturbations to MCM2-7 expression and subcellular distribution reveal exquisite sensitivity of mice to DNA replication stress. *PLoS Genetics*.

[80] Cancer Genome Atlas Research Network (2012). Comprehensive genomic characterization of squamous cell lung cancers. *Nature*.

[81] Honeycutt KA, Chen Z, Koster MI (2006). Deregulated minichromosomal maintenance protein MCM7 contributes to oncogene driven tumorigenesis. *Oncogene*.

[82] Kunnev D, Rusiniak ME, Kudla A, Freeland A, Cady GK, Pruitt SC (2010). DNA damage response and tumorigenesis in Mcm2-deficient mice. *Oncogene*.

[83] Pruitt SC, Bailey KJ, Freeland A (2007). Reduced Mcm2 expression results in severe stem/progenitor cell deficiency and cancer. *Stem Cells*.

[84] Shima N, Alcaraz A, Liachko I (2007). A viable allele of Mcm4 causes chromosome instability and mammary adenocarcinomas in mice. *Nature Genetics*.

[85] Kawabata T, Luebben S, Yamaguchi S (2011). Stalled fork rescue via dormant replication origins in unchallenged S phase promotes proper chromosome segregation and tumor suppression. *Molecular Cell*.

[86] Li XC, Tye BK (2011). Ploidy dictates repair pathway choice under DNA replication stress. *Genetics*.

[87] Bagley BN, Keane TM, Maklakova VI (2012). A dominantly acting murine allele of Mcm4 causes chromosomal abnormalities and promotes tumorigenesis. *PLoS Genetics*.

[88] Steere NA, Yamaguchi S, Andrews CA, Liachko I, Nakamura T, Shima N (2009). Functional screen of human MCM2-7 variant alleles for disease-causing potential. *Mutation Research*.

[89] Hanahan D, Weinberg RA (2000). The hallmarks of cancer. *Cell*.

[90] Negrini S, Gorgoulis VG, Halazonetis TD (2010). Genomic instability—an evolving hallmark of cancer. *Nature Reviews Molecular Cell Biology*.

[91] Jones RM, Mortusewicz O, Afzal I (2013). Increased replication initiation and conflicts with transcription underlie Cyclin E-induced replication stress. *Oncogene*.

[92] Bartkova J, Hořejší Z, Koed K (2005). DNA damage response as a candidate anti-cancer barrier in early human tumorigenesis. *Nature*.

[93] Sun A, Bagella L, Tutton S, Romano G, Giordano A (2007). From G0 to S phase: A view of the roles played by the retinoblastoma (Rb) family members in the Rb-E2F pathway. *Journal of Cellular Biochemistry*.

[94] Ortega S, Malumbres M, Barbacid M (2002). Cyclin D-dependent kinases, INK4 inhibitors and cancer. *Biochimica et Biophysica Acta*.

[95] Fillies T, Woltering M, Brandt B (2007). Cell cycle regulating proteins p21 and p27 in prognosis of oral squamous cell carcinomas. *Oncology Reports*.

[96] Malumbres M, Barbacid M (2001). To cycle or not to cycle: a critical decision in cancer. *Nature Reviews Cancer*.

[97] Sterner JM, Dew-Knight S, Musahl C, Kornbluth S, Horowitz JM (1998). Negative regulation of DNA replication by the retinoblastoma protein is mediated by its association with MCM7. *Molecular and Cellular Biology*.

[98] Pacek M, Walter JC (2004). A requirement for MCM7 and Cdc45 in chromosome unwinding during eukaryotic DNA replication. *The EMBO Journal*.

[99] Doyon Y, Cayrou C, Ullah M (2006). ING tumor suppressor proteins are critical regulators of chromatin acetylation required for genome expression and perpetuation. *Molecular Cell*.

[100] Bellelli R, Castellone MD, Guida T (2014). NCOA4 transcriptional coactivator inhibits activation of DNA replication origins. *Molecular Cell*.

[101] Braden WA, Lenihan JM, Lan Z (2006). Distinct action of the retinoblastoma pathway on the DNA replication machinery defines specific roles for cyclin-dependent kinase complexes in prereplication complex assembly and S-phase progression. *Molecular and Cellular Biology*.

[102] Nallamshetty S, Crook M, Boehm M, Yoshimoto T, Olive M, Nabel EG (2005). The cell cycle regulator p27Kip1 interacts with MCM7, a DNA replication licensing factor, to inhibit initiation of DNA replication. *FEBS Letters*.

[103] Rizwani W, Alexandrow M, Chellappan S (2009). Prohibitin physically interacts with MCM proteins and inhibits mammalian DNA replication. *Cell Cycle*.

[104] Tsao C, Geisen C, Abraham RT (2004). Interaction between human MCM7 and Rad17 proteins is required for replication checkpoint signaling. *EMBO Journal*.

[105] Sherr CJ, Roberts JM (1999). CDK inhibitors: positive and negative regulators of G1-phase progression. *Genes & Development*.

[106] Wang S, Nath N, Adlam M, Chellappan S (1999). Prohibitin, a potential tumor suppressor, interacts with RB and regulates E2F function. *Oncogene*.

[107] Wang S, Nath N, Fusaro G, Chellappan S (1999). Rb and prohibitin target distinct regions of E2F1 for repression and respond to different upstream signals. *Molecular and Cellular Biology*.

[108] Lindsey-Boltz LA, Bermudez VP, Hurwitz J, Sancar A (2001). Purification and characterization of human DNA damage checkpoint Rad complexes. *Proceedings of the National Academy of Sciences of the United States of America*.

[109] Gladden AB, Diehl JA (2003). The cyclin D1-dependent kinase associates with the pre-replication complex and modulates RB-MCM7 binding. *The Journal of Biological Chemistry*.

[110] Shiratori A, Shibata T, Arisawa M, Hanaoka F, Murakami Y, Eki T (1999). Systematic identification, classification, and characterization of the open reading frames which encode novel helicase-related proteins in *Saccharomyces cerevisiae* by gene disruption and Northern analysis. *Yeast*.

[111] Shadrick WR, Ndjomou J, Kolli R, Mukherjee S, Hanson AM, Frick DN (2013). Discovering new medicines targeting helicases: challenges and recent progress. *Journal of Biomolecular Screening*.

[112] Simmons DT (2000). SV40 large T antigen functions in DNA replication and transformation. *Advances in Virus Research*.

[113] Moyer SE, Lewis PW, Botchan MR (2006). Isolation of the Cdc45/Mcm2-7/GINS (CMG) complex, a candidate for the eukaryotic DNA replication fork helicase. *Proceedings of the National Academy of Sciences of the United States of America*.

[114] Luesch H, Wu TYH, Ren P, Gray NS, Schultz PG, Supek F (2005). A genome-wide overexpression screen in yeast for small-molecule target identification. *Chemistry and Biology*.

[115] Glen CD, Dubrova YE (2012). Exposure to anticancer drugs can result in transgenerational genomic instability in mice. *Proceedings of the National Academy of Sciences of the United States of America*.

[116] Woods D, Turchi JJ (2013). Chemotherapy induced DNA damage response: convergence of drugs and pathways. *Cancer Biology and Therapy*.

[117] Guarente L (1993). Synthetic enhancement in gene interaction: a genetic tool come of age. *Trends in Genetics*.

[118] Chan DA, Giaccia AJ (2011). Harnessing synthetic lethal interactions in anticancer drug discovery. *Nature Reviews Drug Discovery*.

[119] Eskander RN, Tewari KS (2014). PARP inhibition and synthetic lethality in ovarian cancer. *Expert Review of Clinical Pharmacology*.

[120] Ibarra A, Schwob E, Méndez J (2008). Excess MCM proteins protect human cells from replicative stress by licensing backup origins of replication. *Proceedings of the National Academy of Sciences of the United States of America*.

[121] Ishimi Y, Sugiyama T, Nakaya R (2009). Effect of heliquinomycin on the activity of human minichromosome maintenance 4/6/7 helicase. *FEBS Journal*.

[122] Toyokawa G, Masuda K, Daigo Y (2011). Minichromosome Maintenance Protein 7 is a potential therapeutic target in human cancer and a novel prognostic marker of non-small cell lung cancer. *Molecular Cancer*.

[123] Liu Y, He G, Wang Y, Guan X, Pang X, Zhang B (2013). MCM-2 is a therapeutic target of Trichostatin A in colon cancer cells. *Toxicology Letters*.

[124] Kwon HJ, Hong YK, Park C (2010). Widdrol induces cell cycle arrest, associated with MCM down-regulation, in human colon adenocarcinoma cells. *Cancer Letters*.

[125] Yun HJ, Hyun SK, Park JH, Kim BW, Kwon HJ (2012). Widdrol activates DNA damage checkpoint through the signaling Chk2-p53-Cdc25A-p21-MCM4 pathway in HT29 cells. *Molecular and Cellular Biochemistry*.

[126] Chino M, Nishikawa K, Umekita M (1996). Heliquinomycin, a new inhibitor of DNA helicase, produced by Streptomyces sp. MJ929-SF2. I. Taxonomy, production, isolation, physico-chemical properties and biological activities. *Journal of Antibiotics*.

[127] Chang J, Varghese DS, Gillam MC (2012). Differential response of cancer cells to HDAC inhibitors trichostatin A and depsipeptide. *British Journal of Cancer*.

[128] Sheu YJ, Kinney JB, Lengronne A, Pasero P, Stillman B (2014). Domain within the helicase subunit Mcm4 integrates multiple kinase signals to control DNA replication initiation and fork progression. *Proceedings of the National Academy of Sciences of the United States of America*.

[129] Oliphant CM, Green GM (2002). Quinolones: a comprehensive review. *The American Family Physician*.

[130] An P, Robles MTS, Pipas JM (2012). Large T antigens of polyomaviruses: amazing molecular machines. *Annual Review of Microbiology*.

[131] Seguin SP, Ireland AW, Gupta T (2012). A screen for modulators of large T antigen’s ATPase activity uncovers novel inhibitors of Simian Virus 40 and BK virus replication. *Antiviral Research*.

[132] Meyer H, Bug M, Bremer S (2012). Emerging functions of the VCP/p97 AAA-ATPase in the ubiquitin system. *Nature Cell Biology*.

[133] Chou T, Brown SJ, Minond D (2011). Reversible inhibitor of p97, DBeQ, impairs both ubiquitin-dependent and autophagic protein clearance pathways. *Proceedings of the National Academy of Sciences of the United States of America*.

[134] Chou T, Li K, Frankowski KJ, Schoenen FJ, Deshaies RJ (2013). Structure-activity relationship study reveals ML240 and ML241 as potent and selective inhibitors of p97 ATPase. *ChemMedChem*.

[135] Firestone AJ, Weinger JS, Maldonado M (2012). Small-molecule inhibitors of the AAA+ ATPase motor cytoplasmic dynein. *Nature*.

[136] Lum PY, Armour CD, Stepaniants SB (2004). Discovering modes of action for therapeutic compounds using a genome-wide screen of yeast heterozygotes. *Cell*.

[137] Giaever G, Flaherty P, Kumm J (2004). Chemogenomic profiling: identifying the functional interactions of small molecules in yeast. *Proceedings of the National Academy of Sciences of the United States of America*.

